# Several occurrences of osteomyelitis in dinosaurs from a site in the Bauru Group, Cretaceous of Southeast Brazil

**DOI:** 10.1002/ar.70003

**Published:** 2025-05-30

**Authors:** Tito Aureliano, Waltécio Almeida, Marcelo A. Fernandes, Aline Marcele Ghilardi

**Affiliations:** ^1^ Department of Biological Chemistry, Programa de Pós‐Graduação em Diversidade Biológica e Recursos Naturais Regional University of Cariri (URCA) Crato Brazil; ^2^ Laboratory of Paleoecology and Paleoichnology Federal University of São Carlos São Carlos Brazil; ^3^ Diversity, Ichnology and Osteohistology Research Group (DINOlab) Federal University of Rio Grande do Norte (URFN) Natal Brazil

**Keywords:** biomineralization, Mesozoic, microanatomy, paleopathology, Sauropoda

## Abstract

This study investigates the occurrence of osteomyelitis in non‐avian dinosaurs, focusing on the Ibirá locality, a site with a high incidence of this pathological condition. We analyzed six new osteopathic sauropod specimens from the Upper Cretaceous of Brazil. The results revealed a relationship between infection and bone remodeling, denoted by various manifestations of reactive bone neoformation, including periosteal reaction. Healing tissues were not identified, which implies that the individuals died when the infection was still active. We described distinct manifestations of osteomyelitis with periosteal bone neoformation: (1) periosteal reaction within small circular protrusions; (2) ellipsoid protrusions in a fingerprint pattern; (3) enlarged protrusions both in height and area. This study underscores the importance of examining pathological conditions in extinct species to enhance our understanding of their physiology and interactions with their ancient environments.

## 1 INTRODUCTION

The study of bone pathology in deep time contexts offers a unique opportunity to assess the evolution of diseases in ancient populations and ecosystems (Chinsamy, 2023; Rothschild et al., 2023a; Rothschild & Martin, 2006). The analysis of fossils from pre‐human times is an ascending field that provides insights into diseases like osteoarthropathy, neoplasia, lytic lesions, and infections (Bertozzo et al., 2023; Cisneros et al., 2010; Ekhtiari et al., 2020; Haridy et al., 2019; Jurmain & Kilgore, 1995; Stilson et al., 2016; Surmik et al., 2022; Xing et al., 2024). Among these diseases, osteomyelitis is commonly reported in the fossil record. Osteomyelitis is a destructive inflammatory condition that results from an osseous infection not restricted to the medulla, which may be bacterial, protozoal, fungal, or viral in etiology (Townsend & Koç, 2023). It may remain localized or expand to involve distinct structures such as the bone marrow, cortex, periosteum, and portions of the surrounding soft tissues (Jorge et al., 2010). This destructive inflammatory condition may progress to osteoblastic bone neoformation, besides sepsis (Smith et al., 2006). The periosteal reaction, also present in some cases of osteomyelitis, consists of the elevation of the cortex, forming new bone as a consequence of the inflammation (Rothschild et al., 2023a). Osteomyelitis has been reported as occurring throughout the Mesozoic in several non‐avian dinosaur clades, including sauropodomorphs, ornithopods, and coelurosaurs (Aureliano et al., 2021; Barbosa et al., 2018; Chinzorig et al., 2022; Cruzado‐Caballero et al., 2023; Hunt et al., 2019; Ramírez‐Velasco et al., 2017; Redelstorff et al., 2015; Tanke & Rothschild, 2002; Woodruff et al., 2022; Xing et al., 2018). Associated causalities for osteomyelitis in the dinosaur fossil record include unhealed fractures and bites (Chinzorig et al., 2022; Xing et al., 2018) and pathogen‐induced infections (Aureliano et al., 2021; Woodruff et al., 2022). Here we report a set of non‐avian dinosaur specimens found within the same site (Ibirá locality, Bauru Group, Southeast Brazil) with the exceptional preservation of different manifestations of osteomyelitis, providing a detailed mosaic of the infection‐driven bone remodeling with and without periosteal reaction.

## 2 MATERIALS AND METHODS

*Institutional Abbreviations*: FMNH, Field Museum of Natural History, Chicago, United States of America; LPP, Laboratory of Paleoichnology and Paleoecology, Federal University of São Carlos, Brazil; MCS‐Pv, Palaeovertebrate collection of the Museo de Cinco Saltos, Río Negro, Argentina; MMNS, Mississippi Museum of Natural Science, Lowndes, United States of America; TMP, Royal Tyrrell Museum of Palaeontology, Drumheller, Canada; UCMP, University of California Museum of Paleontology, Berkeley, United States of America; YX, Yuxi Museum, Yunnan, China.

### 2.1 Specimens

Fossils examined consist of six medium to large‐sized bone fragments. There are indeterminate zygopodial elements (LPP‐PV‐209, 210, 212, 213), “d‐shaped” in the cross‐section view. The medullary cavity is reduced and filled with a trabecular bone matrix, as in all sauropods and different from all theropods, except Spinosaurinae. These resemble the fibula LPP‐PV‐0043 belonging to a specimen of the nanoid saltasaurine *Ibirania parva* (Aureliano et al., 2021; Navarro et al., 2022). Therefore, LPP‐PV‐210, 212, and 213 are tentatively interpreted as indeterminate sauropod zygopodia.

LPP‐PV‐211 and 214 comprise elongated bones, flattened laterally. The medullary cavity is also filled with trabecular bone. Therefore, these elements are interpreted herein as indeterminate sauropod ribs. However, the size difference between both specimens is worth noting, which suggests they belonged to two distinct organisms.

### 2.2 Locality and horizon

The fossils were collected during several academic excursions to the “Vaca Morta” Site (Municipality of Ibirá, São Paulo state backcountry) between 2006 and 2023. They were collected over the surface and subsurface of a hillside within the São José do Rio Preto Formation, Upper Cretaceous (Santonian), Bauru Group (see the stratigraphic description of the site in the supplementary material of Navarro et al., 2022). The collectors had no control over which exact stratum each piece came from, but it is certain that the fossils came from similar layers in a vertical range of 2 m. These are the same layers that provided the *Ibirania* specimens (Navarro et al., 2022).

### 2.3 Photography

Images were taken with a Leica MC170 HD video camera attached to a Leica M205C with a Planapo 0.5 or 1.0× objective. Figures were produced from stacks of images using LAS (Leica Application Suite) v4.12. Image tables were elaborated in Adobe Illustrator CC 2024.

### 2.4 Paleopathology

We follow Rothschild and colleagues in describing paleopathologies (Rothschild et al., 2023a, 2012; Rothschild & Martin, 2006). Here, we follow the definition of osteomyelitis as an infection‐driven bone lesion that is not limited to the surface (Rothschild et al., 2012).

## 3 RESULTS

**FIGURE 1:**
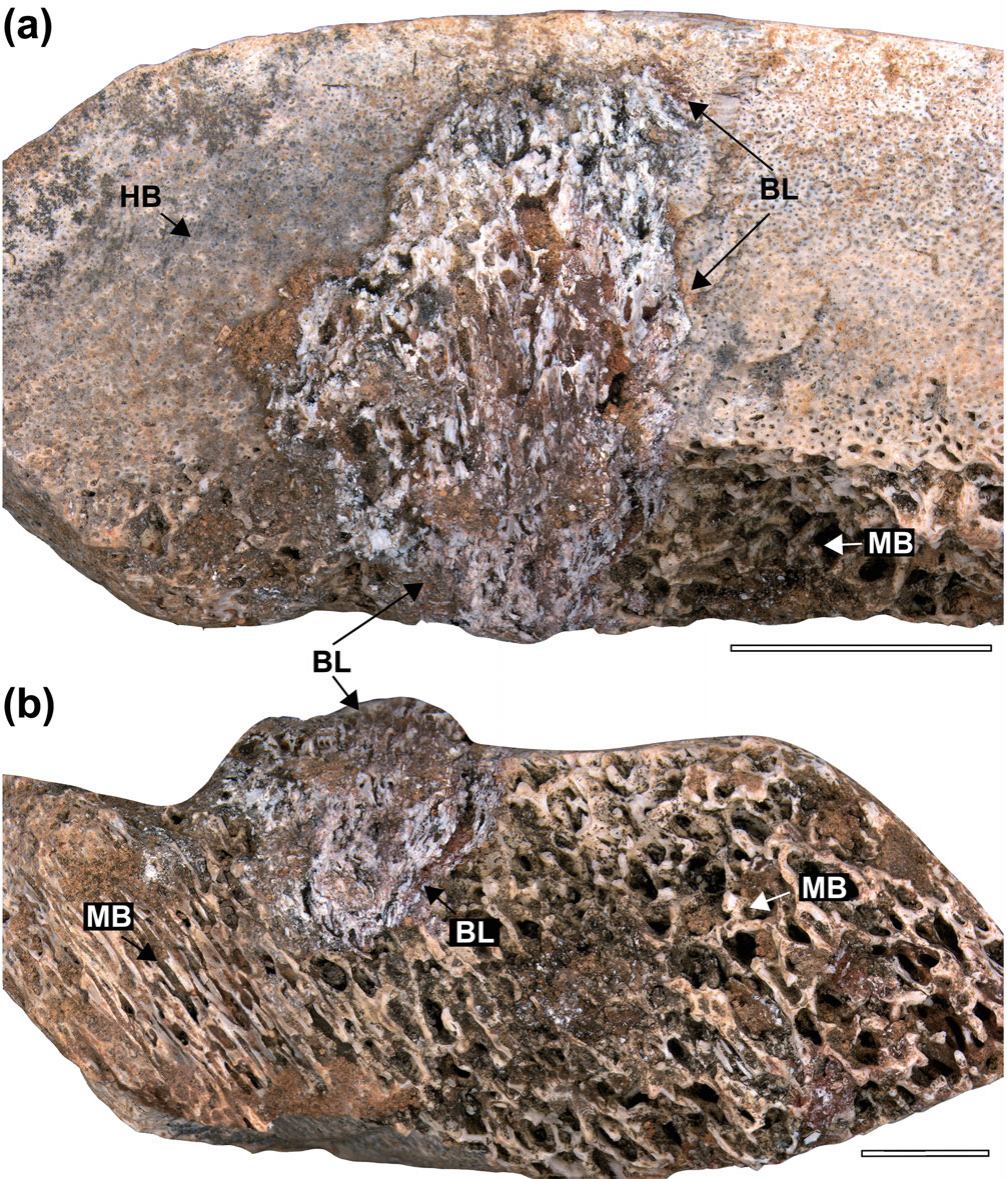
Non‐protruding osteomyelitis in a sauropod zygopodial element (LPP‐PV‐209). The cross‐section in (a) Longitudinal view in (b) BL, bone lesion; HB, harversian bone; MB, medullary bone. Photomicrography conducted by Luciana B. R. Fernandes. Scale bar in a = 5 mm; in b = 3 mm.

**FIGURE 2:**
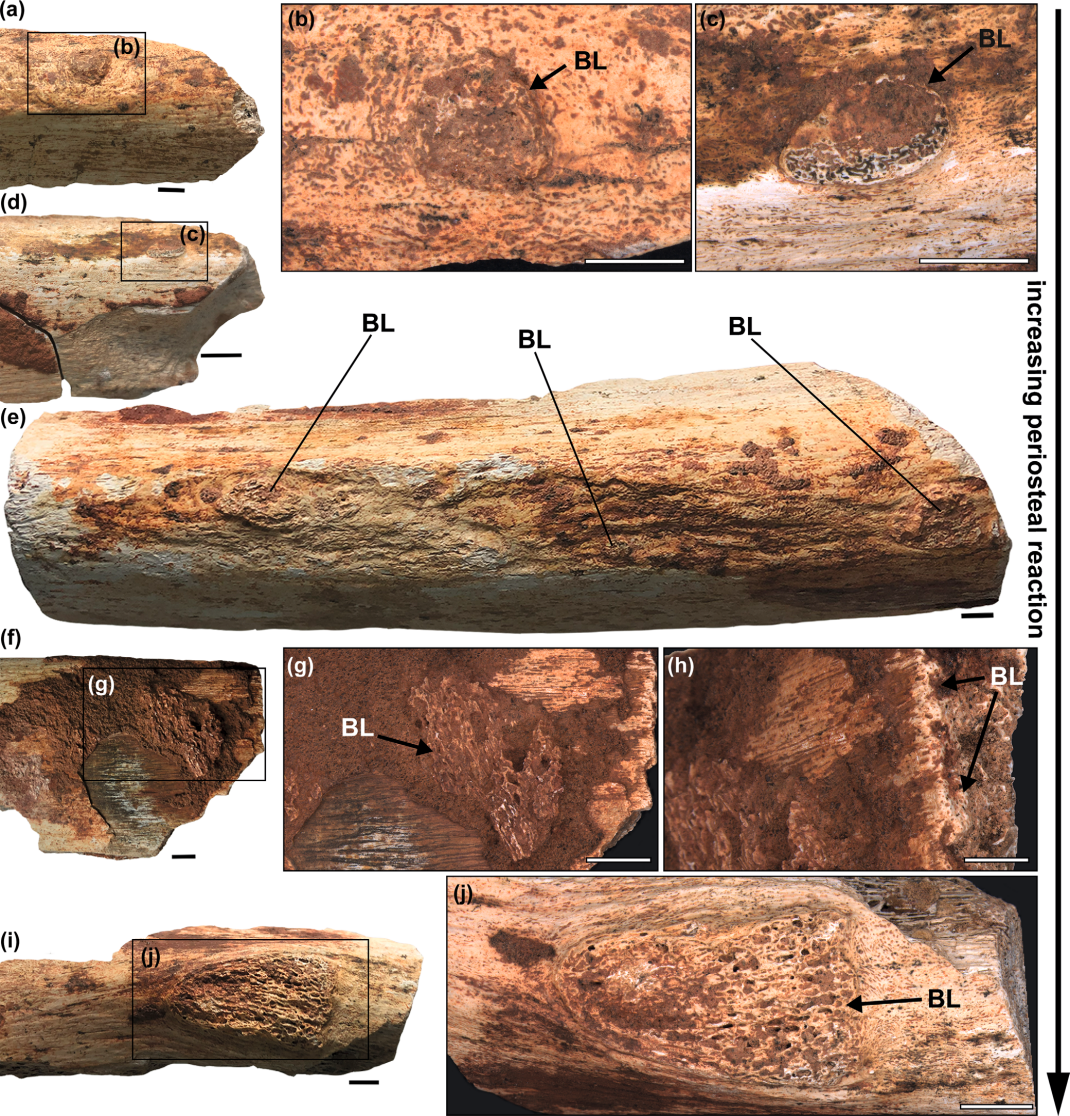
Protruding osteomyelitis in sauropod zygopodia (a)–(d), (i), (j) and ribs (e), (f)–(h). Note the increasing periosteal reaction from (a) to (j). Specimens imaged: LPP‐PV‐213 (a), (b), LPP‐PV‐212 (c), (d), LPP‐PV‐211 (e), LPP‐PV‐214 (f)–(h), and LPP‐PV‐210 (i), (j). BL, bone lesion. Photomicrography conducted by Luciana B. R. Fernandes. Scale bar in a, e, f, i = 5 mm; in b, c, g, h, j = 3 mm; in d = 10 mm.

Despite the fragmentary nature of the specimens, the taphonomic aspects allowed the excellent preservation of the pathologies (Figures 1 and 2). Lytic lesions or bite marks are absent; all fractures have post‐mortem taphonomic effects. There is no abraded cortex on the surface of most specimens (LPP‐PV‐209 to 213). There are signs of cortical abrasion in LPP‐PV‐214, but those areas do not include lesions, and the underlying bone microtexture differs from the reactive periosteum described in sequence (Figure 2f, h). All specimens present bone lesions, and all but LPP‐PV‐209 (Figure 1) present protruding periosteal reactions (Figure 2). Lesions in all specimens present a spongy tissue architecture (a chaotic trabecular architecture) within delimited zones in the cortex. Those that protrude throughout the surface occur in a “fingerprint” sequence (sensu Rothschild & Martin, 2006). However, there is a diverse spectrum in the pattern in which these periosteal reactions occur: some are smaller and rounded (Figure 2a, b), and others present a larger ellipsoid format with higher relief (Figure 2c, j). This differs from the granular or spicular textures observed in bone neoplasia and osteosarcoma, and the granular texture seen in tuberculosis‐like lesions (Cruzado‐Caballero et al., 2021; Ekhtiari et al., 2020; Rothschild & Martin, 2006; Surmik et al., 2018). However, since both of the latter can also manifest in an osteolytic manner, texture alone could not be a distinguishing characteristic for diagnosis. The fingerprint pattern present on the diaphyses of zygopodia and ribs differs from the reactions present in osteoarthropathy, which are restricted to articulation zones (Jurmain & Kilgore, 1995; Rothschild et al., 2023b; Rothschild & Martin, 2006). These lesions are dome‐shaped in cross‐section (see BL in Figure 1a) and connect the medulla to the outer cortex (Figure 1a, b and Figure 2h). These characteristics and the elements affected (zygopodia and ribs) suggest the diagnosis of osteomyelitis for the pathologies in all specimens (Rothschild et al., 2023a, 2012).

Non‐pathological linear cortical striations have also been observed on the bone surface (Figure 2a, d–j). These represent a normal growth phenomenon of non‐reactive periosteum (Rothschild et al., 2023c).

## 4 DISCUSSION

The reactive bone described in LPP‐PV‐209 to 214 decreases in bone volume density (area covered by bone tissue versus area covered by vascular canals + resorption cavities) compared to the nearby cortical tissue. This condition was noted in the pathological fibula of *Ibirania parva* LPP‐PV‐0200, diagnosed with acute osteomyelitis (Aureliano et al., 2021), and contrasts with the increase of bone density often seen in osteosarcoma and tuberculosis‐like lesions (Surmik et al., 2018). This highly vascularized bone tissue results from the osteoblastic bone neoformation in an intensive osteogenic scenario (Aureliano et al., 2021; Huttenlocker et al., 2013; Rothschild et al., 2023a). However, no signs of abscess and sequestrum were found on the Ibirá pathological sauropods, contrary to those observed in the post‐traumatic infection in a rib of *Lufengosaurus* YX V0003 (Xing et al., 2018) and the ulna of *Dilophosaurus* UCMP 37302 (Senter & Juengst, 2016). Additionally, all manifestations of periosteal reaction in LPP‐PV‐210 to 214 are focal and not continuously widespread across the periosteal surface as seen in some hadrosaurs TMP 1980.016.0162 and TMP 2008.012.0066 (Tanke & Rothschild, 2014), and coelurosaurians FMNH PR2081 and MMNS VP‐6332 (Chinzorig et al., 2022; Hamm et al., 2020). Our specimen with the most aggressive reactive bone foci (LPP‐PV‐210) still demonstrates more subtle protrusions than the ones seen in the titanosaurs LPP‐PV‐200 (Aureliano et al., 2021) and MCS‐PV 183/2 (García et al., 2017).

Several cases of healing injuries have been described from ceratopsians, ornithopods, and theropods (Bertozzo et al., 2024; Cruzado‐Caballero et al., 2021; Lü et al., 2007; Senter & Juengst, 2016; Xing et al., 2024). Healing or healed bone tissue caused by trauma is detected from a callosity present at the site (Hao et al., 2021; Rothschild et al., 1993). Surprisingly, none of the osteopathic sauropod specimens from the Ibirá Site show any evidence of healing tissues, and the lesions were still active at the time of their death. Finally, the pathological specimens are absent of common osteomyelitis structures like involucrum, sequestrum, and cloacae, and hence, this implies a relatively fast advance of the disease (Rothschild et al., 2023a; Rothschild & Martin, 2006).

### 4.1 Different manifestations of bone remodeling in osteomyelitis

**FIGURE 3:**
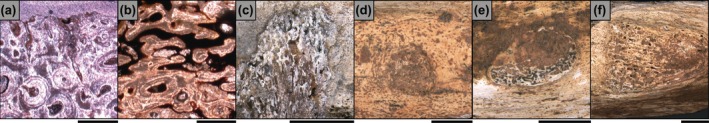
Histopathologic development in osteomyelitis. (a), (b), LPP‐PV‐0043 (Aureliano et al., 2021); polarized light with crossed (a) and parallel (b) nicols. (c), LPP‐PV‐0209. (d), LPP‐PV‐0213. (e), LPP‐PV‐0212. (f), LPP‐PV‐0210. Diagram built with SankeyMATIC (Bogart, 2014). Scale bar in a = 200 microns; in b = 500 mic; c = 5 mm; d–f = 3 mm.

Aureliano et al. (2021) suggested different histological presentations of osteoblastic bone neoformation over the periosteum along the advancement of acute osteomyelitis before necrosis in the saltasaurine titanosaur *Ibirania*. Here, we described several additional specimens with varied degrees of infection‐driven bone remodeling, with and without periosteal reactions. In LPP‐PV‐209 (Figure 1), the osteomyelitis lesion originates from the endosteal area and develops toward the outer cortex, but does not reach the periosteum. Osteomyelitis lesions are not limited to growing from the medulla toward the periosteum, but could also advance and spread the other way around (Rothschild et al., 2023a). In the rib of LPP‐PV‐214, the focal reactive bone is limited to the periosteum and the outer cortex (Figure 2h). Nonetheless, here we describe different manifestations of the periosteal bone neoformation observed in our specimens and suggest a mosaic of distinct presentations of the disease (Figure 3), listed below:

- One group consists of periosteal reactions that consist of small circular protrusions (see LPP‐PV‐213; Figure 2a, b).
- Some protrusions appear in a fingerprint pattern and each one attains an ellipsoid shape (LPP‐PV‐211,212; Figure 2c–e).
- There are enlarged protrusions that enlarge and increase both in relief and area (see LPP‐PV‐214,210; Figure 2f, j).

## 5 CONCLUSION

We provided insights into the prevalence of osteomyelitis among non‐avian dinosaurs, particularly from a notable site from the Upper Cretaceous of Southeast Brazil. We documented distinct manifestations of reactive bone neoformation with and without periosteal reaction, revealing a complex relationship between infection and bone remodeling. In this sense, the osteomyelitis rapidly advanced in the specimens with no protusions and a bit slower in the individuals with protusions in the absence of sequestrum, involucrum, and cloacae. These infections were active at the time of death, potentially affecting the organisms' overall health and survival. This study enhances our understanding of the impact of disease on dinosaur physiology and underscores the importance of studying pathological conditions in extinct species to gain insights into their life histories and interactions with their environments.

## AUTHOR CONTRIBUTIONS

**Tito Aureliano:** Conceptualization; investigation; funding acquisition; writing – original draft; methodology; validation; visualization; writing – review and editing; software; formal analysis; project administration. **Waltécio Almeida:** Conceptualization; funding acquisition; writing – review and editing; methodology; formal analysis; supervision; project administration. **Marcelo A. Fernandes:** Investigation; writing – review and editing; validation; data curation; supervision; resources. **Aline Marcele Ghilardi:** Conceptualization; investigation; funding acquisition; writing – review and editing; validation; methodology; data curation; resources.

## CONFLICT OF INTEREST STATEMENT

The authors declare no competing interests.

## Data Availability

All fossils are housed in a research institution and can be accessed through a request to the collection's curator (M.A.F.).
